# Supplementary material for: Adolescents and young adults are the most undiagnosed of HIV and virally unsuppressed in Eastern and Southern Africa: Pooled analyses from five population-based surveys
Source: PLOS Glob Public Health. 2023 Dec 22;3(12):e0002398. doi: 10.1371/journal.pgph.0002398 (PMC10745138; doi:10.1371/journal.pgph.0002398)
Supplement: S1 Text — (DOCX) [file pgph.0002398.s001.docx]

**Supplementary methods**

**Study sites**

In Kenya, the rural sub-county of Ndhiwa (190,000 inhabitants, census 2012), is located in Homa-Bay County, the area of Kenya most affected by HIV. In 2001, Médecins Sans Frontières (MSF) and the Ministry of Health (MOH) initiated an HIV/AIDS program in Homa Bay County including HIV testing and counselling and antiretroviral therapy (ART) at hospital level. Later, ART was decentralized to the peripheral centers in the county. The HIV program included patients’ care and monitoring, diagnosis and treatment of opportunistic diseases, treatment adherence counselling, social support, pediatric HIV care, prevention of mother to child transmission (PMTCT), family planning, as well as training and mentoring of health staff in peripheral facilities. The study took place in 2012, prior to MSF engaging specifically in Ndhiwa sub-county.

In South Africa, the city of Eshowe and the surrounding rural area of Mbongolwane are in the municipality of uMlalazi Municipality, province of Kwazulu Natal. The survey was conducted in 14 administrative wards (120,000 inhabitants, census 2011) of the municipality. In 2011, MSF in partnership with the Department of Health (DOH) launched a community-based “Treatment as Prevention” pilot project including HIV prevention activities such as large-scale community-based HIV counselling and testing (door-to-door and at fixed community testing sites), condom distribution, voluntary medical male circumcision and health promotion activities. In addition, the project supported decentralized ART initiation, adherence counselling training and mentoring of health staff in peripheral facilities and implementation of the national adherence and clinical guidelines, including scale-up of viral load monitoring, management of treatment failure and TB/HIV integration. The survey was conducted in 2013, prior to the full-scale implementation of the HIV program.

In Malawi, the rural district of Chiradzulu (270,000 inhabitants, census 2012) is in the southern part of the country. In 1997, MSF initiated an HIV/AIDS program in this area, through medical care, treatment of opportunistic diseases, and palliative care. In August 2001, the fixed dose combination of tri-therapy was introduced. By 2004, ART was decentralized to 10 peripheral health centers. The same year, a “task-shifting program” was launched for nurses to follow-up stable patients on ART first, and later to initiate ART. In 2013, the year of the survey, a large number of HIV services were implemented by the MOH and MSF: HIV counselling and testing, HIV adult and pediatric care, ART delivery, PMTCT, treatment adherence counselling, health education, tuberculosis diagnosis and treatment, and voluntary male medical circumcision.

The rural district of Nsanje (300,000 inhabitants, census 2018), in Malawi, is also in the most southern region of the country and is one of the poorest. Since 2011, MSF and the MoH were involved in HIV/TB care with a decentralization process of ART. Activities such as HIV testing services PMTCT, community ART refill groups, health education, tuberculosis diagnosis and treatment, voluntary male medical circumcision were progressively implemented over the years. In 2013, routine viral load testing on dried blood spot was implemented in the 14 health centers of the district. In 2016, the year of the survey, the new ART initiation guidelines including “treat all” recommendation, were implemented.

In Zimbabwe, the district of Gutu, (200,000 inhabitants, census 2012), is located in the Maswingo Province, in the South-Eastern part of the country. In 2002, MSF started supporting the MOH HIV/TB programs. In 2004, MSF initiated an ART program in the clinic located in the Murambinda Mission Hospital of Buhera district. ART delivery decentralization was organized in peripheral facilities and a “task-shifting program” was started. As many people from neighboring districts attended the mobile clinic, MSF extended its program to Gutu district in 2011. ART delivery, staff mentoring, implementation of new ART initiation guidelines and routine viral load were part of the decentralization of HIV care. In 2013, 31 health facilities of this district delivered ART. In 2015, activities included ART delivery, PMTCT, counselling, differentiated model of care including community ART groups, viral load monitoring, health education, tuberculosis diagnosis and treatment and voluntary male medical circumcision. In 2016, the year of the survey, the new ART initiation guidelines including “treat all” recommendation, were implemented.

**Study procedures**

In KwaZulu-Natal, HIV anonymous testing in the laboratory was also offered to the participants. Viral load measurement testing was performed using a COBAS Amplirep/Cobas Taqman platform in Kenya (Roche Diagnostic System, Branchburg, New Jersey, USA), G2 real-time PCR in Chiradzulu, Malawi (Biocentric®, Bandol, France), NucliSens EasyQ HIV-1 v2.0 in South Africa (Biomerieux, Lyon, France), HIV RNA PCR (Abbott RealTime HIV-1 platform (m2000sp), USA) in Nsanje, Malawi and dried blood sport (Nuclisens HIV-1 QT assay BioMerieux®, Marcy-Etiole, Rhône) in Zimbabwe.

**Ethics review boards**

- Ethics Review Board instituted by Médecins Sans Frontières (MSF ERB) – References 1619 and 1622
- French ethical committee “Comité de Protection des Personnes d’Ile de France” – References 12056, 12084 and 120191
- Kenya Medical Research Institute Ethical Review Committee (KEMRI) – Reference 347
- University of Cape Town Human Research Ethics Committee (HREC) – Reference 461/2012
- Health Research Committee of the Health Research and Knowledge Management Unit of KwaZulu-Natal Department of Health – Reference HRKM-008/13
- The Medical Research Council of Zimbabwe - Reference MRCZ/A/2075
- The National Health Sciences Review Committee of Malawi – References 1085 and 1598
