# Supplementary material for: Adolescents and young adults are the most undiagnosed of HIV and virally unsuppressed in Eastern and Southern Africa: Pooled analyses from five population-based surveys
Source: PLOS Glob Public Health. 2023 Dec 22;3(12):e0002398. doi: 10.1371/journal.pgph.0002398 (PMC10745138; doi:10.1371/journal.pgph.0002398)
Supplement: S1 Table — (DOCX) [file pgph.0002398.s002.docx]

**S1 Table: Description of the study sites and the HIV program activities in the surveyed areas at the time of the survey**

|  | Ndhiwa,  Kenya  (2012) | Eshowe,  South Africa  (2013) | Chiradzulu,  Malawi  (2013) | Nsanje,  Malawi  (2016) | Gutu,  Zimbabwe  (2016) |
| --- | --- | --- | --- | --- | --- |
| Population | 190,000 | 120,000 | 270,000 | 300,000 | 200,000 |
| Year of initiation MSF support | 2001 | 2011 | 1997 | 2016 | 2012 |
| ART treat all guidelines at the time of the survey | No | No | No | Yes | Yes |
| HIV testing and counselling | Yes | Yes | Yes | Yes | Yes |
| ART delivery at hospital | Yes | Yes | Yes | Yes | Yes |
| ART delivery decentralized at peripheral facilities | Yes | Yes | Yes | Yes | Yes |
| Nurse-initiated and management of ART | Yes | Yes | Yes | Yes | Yes |
| CD4 monitoring | Yes | Yes | Yes | Yes | Yes |
| Viral load monitoring | Yes | Yes | No | Yes | Yes |
| Treatment adherence counselling | Yes | Yes | Yes | Yes | Yes |
| Management opportunistic infections | Yes | Yes | Yes | Yes | Yes |
| Prevention of mother to child transmission | Yes | Yes | Yes | Yes | Yes |
| Voluntary male circumcision | Yes | Yes | Yes | Yes | Yes |
| Condom distribution | Yes | Yes | Yes | Yes | Yes |
| Health promotion activities | Yes | Yes | Yes | Yes | Yes |
| Social support | Yes | Yes | No | Yes | Yes |
