# Supplementary material for: Adolescents and young adults are the most undiagnosed of HIV and virally unsuppressed in Eastern and Southern Africa: Pooled analyses from five population-based surveys
Source: PLOS Glob Public Health. 2023 Dec 22;3(12):e0002398. doi: 10.1371/journal.pgph.0002398 (PMC10745138; doi:10.1371/journal.pgph.0002398)
Supplement: S2 Table — (DOCX) [file pgph.0002398.s003.docx]

**S2 Table: Median age by gender of the participants included in the pooled analyses in the 5 surveyed sites**

|  | **Ndhiwa,**  **Kenya** | **Eshowe, South Africa** | **Chiradzulu,**  **Malawi** | **Nsanje,**  **Malawi** | **Gutu,**  **Zimbabwe** | **Overall** |
| --- | --- | --- | --- | --- | --- | --- |
| **Included in pooled analyses (N)** | **6076** | **5649** | **7269** | **4016** | **3733** | **26740** |
| All, median years [IQR] | 29 [20-40] | 26 [19-40] | 29 [20-39] | 30 [21-40] | 32 [21-43] | 29 [20-40] |
| Women, median years [IQR] | 29 [22-40] | 28 [20-41] | 29 [21-39] | 30 [22-40] | 34 [23-44] | 30 [21-41] |
| Men, median years [IQR] | 29 [20-41] | 28 [20-43] | 28 [19-38] | 30 [20-41] | 28 [19-42] | 27 [19-40] |
| **HIV positive (N)** | **1457** | **1423** | **1233** | **525** | **583** | **5221** |
| All, median age [IQR] | 34 [27-43] | 34 [27-42] | 36 [31-44] | 37 [30-45] | 41 [33-47] | 36 [28-44] |
| Women, median age [IQR] | 31 [25-40] | 33 [26-42] | 35 [30-43] | 36 [30-43] | 40 [33-47] | 35 [27-43] |
| Men, median age [IQR] | 38 [31-47] | 36 [29-44] | 39 [33-45] | 40 [33-47] | 42 [34-47] | 39 [31-46] |
