# Supplementary figures and images for: Adolescents and young adults are the most undiagnosed of HIV and virally unsuppressed in Eastern and Southern Africa: Pooled analyses from five population-based surveys
Source: PLOS Glob Public Health. 2023 Dec 22;3(12):e0002398. doi: 10.1371/journal.pgph.0002398 (PMC10745138; doi:10.1371/journal.pgph.0002398)

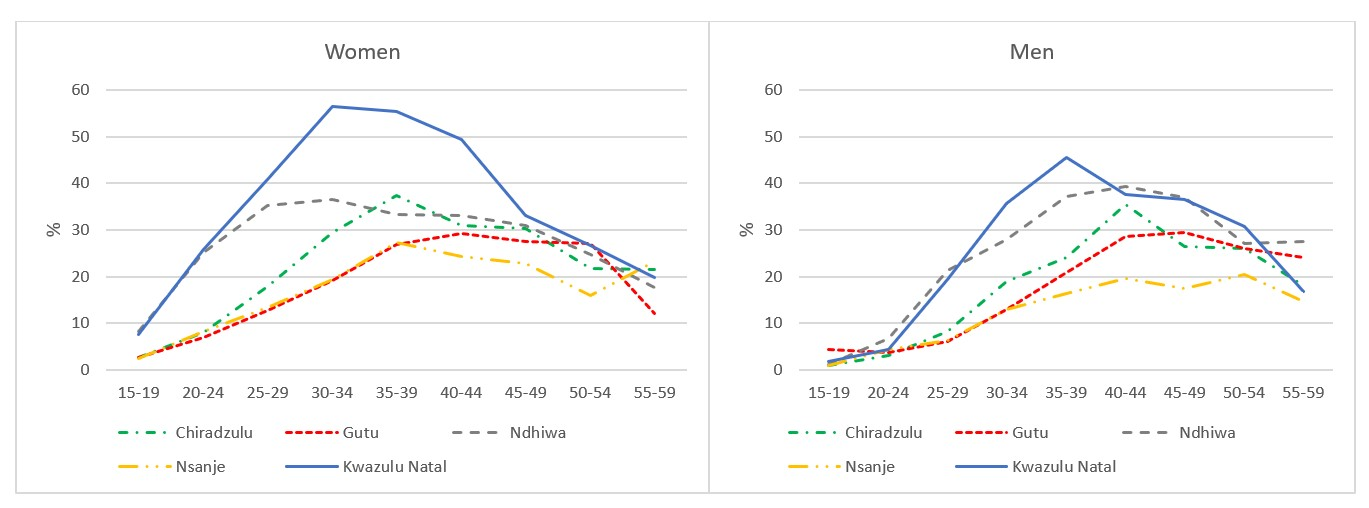

Supplement: S1 Fig — (TIFF) [file pgph.0002398.s004.tiff]

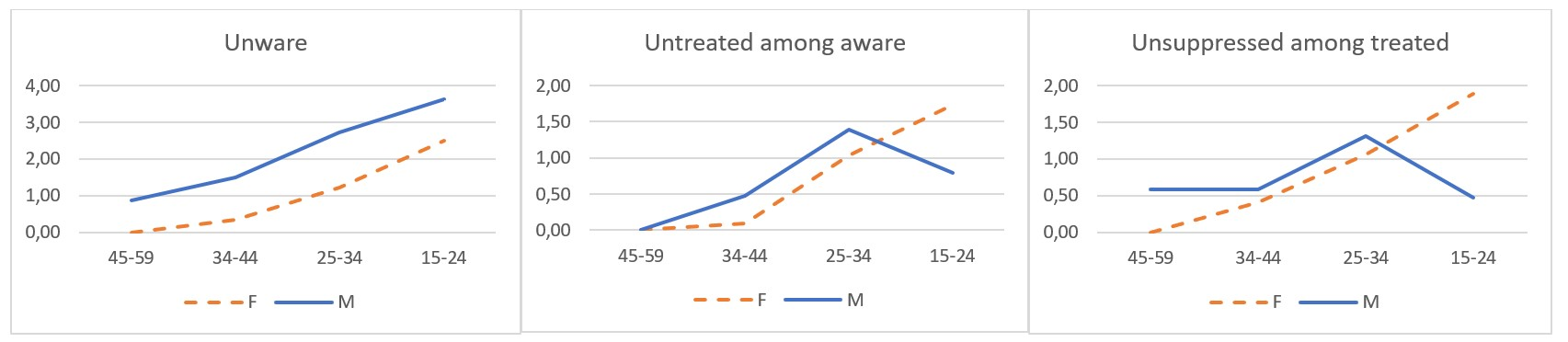

Supplement: S2 Fig — (TIFF) [file pgph.0002398.s005.tiff]
