# Supplementary material for: Adolescents and young adults are the most undiagnosed of HIV and virally unsuppressed in Eastern and Southern Africa: Pooled analyses from five population-based surveys
Source: PLOS Glob Public Health. 2023 Dec 22;3(12):e0002398. doi: 10.1371/journal.pgph.0002398 (PMC10745138; doi:10.1371/journal.pgph.0002398)
Supplement: Attachment — Submitted filename: Response to Reviewers.docx [file pgph.0002398.s006.docx]

PGPH-D-23-00391
Adolescents and young adults are the most undiagnosed of HIV and virally unsuppressed in Eastern and Southern Africa: pooled analyses from five population-based surveys.
PLOS Global Public Health

Reviewer #1: This is a well written paper even though for the four sites, study was conducted before the latest WHO eligibility guidelines were implemented.
However, you mentioned that viral load measurement was performed regardless of ART status, it would be great to also see the breakdown of the VLS status for the undiagnosed study participants especially since your study title speaks to the undiagnosed and non-virally suppressed.

**Response: Thank you very much for your review of our manuscript. We hope to have addressed the points you’ve raised in the revised version of the manuscript.**

**The VL measurement was performed for all the study’s participants indeed. We have changed accordingly the sentence in the Methods to avoid any confusion.**

***“Viral load measurement was performed for all individuals who tested positive for HIV, regardless of their ART status.”* Methods: page 7, line 117-118**

**Regarding the title of the manuscript and the analyses done, we have focused our analyses on the HIV diagnostic cascade (diagnosed, treated, virally suppressed). The title of the manuscript refers to the first and last steps of the cascade. We have carefully considered the additional analysis proposed and we thought that these additional results would go out of our initial focus on the HIV cascade. Thanks again for this suggestion.**

Reviewer #2: My review has been added as an attachment [Editor note: copied below instead of attached]; however, I would add that the paper is an important addition to the literature on the treatment cascade of adolescents and young adults spanning 5 settings in four countries, and with a very large sample size. The attached comments should therefore not negate the contribution this piece will make to our knowledge on the subject.

**Response: Thank you for your thoughtful review of our manuscript. We hope to have addressed the points you’ve raised in the revised version of the manuscript.**

**Abstract**:

- Line 47 - 72.8% etc were diagnosed – previously or newly diagnosed? Not clear in abstract but becomes clear in the methods; some readers do not go beyond abstract

**Response: Thank you for this comment. We hope to have clarified this in the revised manuscript.**

***“Of them, 72.8% were previously diagnosed and 56.7% virally suppressed (88.5% among those treated).”.* Abstract: page 3, Line 48**

- Line 61 – treating or treatment?

**Response: Thanks. We have changed to treatment.**

**Introduction**:

- Lines 71-72 – May want to stick to the focus of the paper – AYA

**Response: Thanks for the suggestion. We have changed the focus to the AYA group. Introduction: Page 5, Lines 75-81**

- Lines 72-74 - Make the case for age differentials first - the focus of the paper (and title), then by gender

**Response: Thank you for this valuable comment. We have changed accordingly in the revised manuscript.**

***“There are reports showing poorer outcomes on the HIV testing and treatment cascade (including the 90-90-90 indicators) at younger ages (2,8–12). However, a large study conducted in four low-income countries did not find an effect of young age on HIV testing (13). There is also substantial evidence of a gender differential within the HIV cascade, with men having lower rates of HIV diagnosis and treatment and higher mortality (2–5). Designing specific interventions to address gender inequities has been recommended but translation into operational programs on a large scale is still pending (6,7). “* Introduction: Page 5, line 75-81**

- Lines 75-71 - Provide background of AYA vs older adults in the 3 key outcomes in the HIV treatment cascade, not just testing

**Response: Thanks for the comment. We have clarified this in the background.**

***“There are reports showing poorer outcomes on the HIV testing and treatment cascade (including the 90-90-90 indicators) at younger ages.”***

- Line 77 - Gender does not come out in the title so a reader is led to believe the paper is just about age

**Response: Thank you very much for your valuable comment. We appreciate your attention to the title of our article and understand the concern raised. Indeed, gender inequality in the cascade of care for HIV is an important aspect to consider, and it was one of the hypotheses we aimed to explore in our study. However, upon conducting the pooled analyses, we found a particularly striking and concerning trend related to adolescents and young adults (AYA) in the context of HIV diagnosis and viral suppression. While gender inequality remains a pertinent issue in HIV care and prevention, we decided to prioritize highlighting the critical concern surrounding AYA based on the data and its potential impact on public health outcome, in the title.**

- Overall, the background does not convincingly or clearly make the case for the paper. Need to tighten this to make the reading flow

**Response: We have modified the background to make clearer the case for the paper. Page 5, lines 82-86**

- Somewhere under Study Design, consider stating the objective(s) of the analysis in this section

**Response: Thank you for your comment, we stated the objectives in the Methods section. *“To evaluate the HIV diagnosis and treatment coverage and identify the age-gender groups with the highest proportions of unfavorable outcomes in the HIV cascade, three main indicators were estimated for the HIV-positive individuals.”* Methods: page 7, line 124-127**

- Line 106 - respective national guidelines to indicate it's not a uniform guideline in all counties

**Response: Thank you, we have changed accordingly in the revised manuscript.**

***“All participants were tested for HIV at home by certified lay counsellors using a serial rapid testing algorithm according to respective national guidelines and included”.* Manuscript: page 7, line 116.**

- Lines 114-115 - This is explained here but not clear in abstract

**Response: Thank you for your valuable comment, we have clarified in the abstract**

**“The HIV cascade outcomes and the risk of being undiagnosed, untreated among those diagnosed, and virally unsuppressed (≥1,000 copies/mL) among those treated were assessed for several age-gender groups. *“.* Abstract: Page 3, line 43-45**

- Lines 116-119 – Number doesn't tell much. consider just proportion

**Response: Thank you, we have changed accordingly.**

**Results**:

- Lines 209-2013 - Shouldn't this summary be about those undiagnosed. Confusing

**Response: The summary about those undiagnosed was in Lines 197-201 in the first version of the manuscript. Currently this is in Lines 211-215 in the tracked version.**

- Overall, consider also comparing viral load between diagnosed and undiagnosed by age group so we see entry point by age – e.g., are  AYA still performing worse? Another analysis that would be on interest is comparing AGYW with ABYM.

**Response: Thank you for your suggestion. We assessed viral load among diagnosed and treated and among all HIV-positive by age group (Table 3). We found that among all HIV-positive, viral suppression was lower among adolescents and young adults than among elder individuals. However, we have not performed the specific breakdown comparing viral load among diagnosed and undiagnosed as we have focused our analysis on the HIV cascade of care. We expected that most of the patients diagnosed and untreated were virally unsuppressed and most of the patients undiagnosed were also virally unsuppressed.**

**We have performed an additional analyse comparing viral suppression in AGYW and ABYM as suggested. We have included the results in the Results section, page 12, lines 199-201 and 210-213.**

***“Adolescent girls and young women (15-24 years) on ART were less virally suppressed than adolescent boys and young men (15-24 years):  75.4% (132/175) vs 90.9% (30/33), p= 0.05.”***

***“There was no difference by gender in the proportion of viral suppression in adolescent girls and young women, and adolescent boys and young men:  32.7% (177/541) vs 33.6% (38/113), respectively, p= 0.85.***

**Discussion**:

- Lines 218-219 - important reinforcement of risk of AYA

**Response: Thank you for your comment. We, indeed, have developed this aspect in the discussion.**

- Line 234 -life/lives?

**Response: Thank you for your comment. We have changed this.**

- Line 238 - sounds hanging. consider adding .. young 'women'

**Response: Thank you for your comment, we have changed the sentence.**

***“As HIV prevalence in women is higher than in men, an important proportion of the overall number of individuals undiagnosed were women, mostly young women”.* Manuscript: page 15, line 259.**

- Line 263 - do not (not don’t)

**Response: Thanks. We have changed.**

- Line 269 - …including untreated patients… in discussion. Besides reporting combined data occasionally, the results are mostly on diagnosed and very little in undiagnosed = untreated. See other comments above

**Response: Thank you very much for your valuable comment and insightful observations. We have removed the part of the sentence referring to untreated patients.**

- Lines 275-276 - Repeated - already stated in 1st part of this sentence

**Response: We have removed the sentence.**

- Line 276 -  “Therefore, interventions adapted to these groups should be urgently enhanced”  Sentence does not add value – consider dropping

**Response: Thank you. We have dropped the sentence.**

Reviewer #3: This is an important study given the increasing numbers of adolescents living with HIV and continued gender and age disparities along the HIV continuum of care. The authors attempt to address this critical time by conducting a pooled data analyses of population-based surveys in Kenya, South Africa, Malawi, and Zimbabwe to evaluate for gender- and age-specific trends along the HIV continuum of care. The study found that adolescents and young adults ages 15-24 years old were less likely to know their HIV diagnosis, be on treatment, and virally suppressed compared to older age groups.

Overall, the interpretation of the findings are good. The major strengths of this manuscript are the focus on a population (adolescents and young adults living with HIV in Sub-Saharan Africa) that has high rates of morbidity and mortality at each point of the continuum of care, as well as a large sample size with HIV testing and viral load testing, that supports your discussion. However, a few additional points to consider:

**Response: Thank you for your thoughtful review of our manuscript. We hope to have addressed the points you’ve raised in the revised version of the manuscript.**

1. Introduction: Good overview of gender/age disparities along HIV continuum of care, however I would add in a few sentences about why you chose to study these particular geographic regions (add in some more epidemiological information about HIV epidemic in Sub-Saharan Africa)

**Response: We have modified the background and included that the study focused on high HIV prevalence settings. Background, Page 5, Lines 84-86**

2. Methods: Your statistical methods are well described. However, please expand on what you mean by “In KwaZulu-Natal ART testing in blood was also performed” (line 108). Did you check for ART resistance? Did you for specific drug levels of medications?

**Response: Thank you very much for your comment. We have clarified in the text that in KZN, qualitative testing for detection of antiretrovirals was performed. We have also added the reference reporting details on the testing done.**

**Methods, Page 7, lines 118-119**

**We also performed ART resistance testing in the survey conducted in KZN. However, we did not include the resistance results in this paper as testing was not performed in all sites. The specific results of the survey in KZN have been published in the referenced article: Huerga H, Van Cutsem G, Ben Farhat J, et al. Progress towards the UNAIDS 90-90-90 goals by age and gender in a rural area of KwaZulu-Natal, South Africa: A household-based community cross-sectional survey. BMC Public Health, 2018.**

3. Discussion: Please expand on past studies that highlight cabotegravir for HIV prevention in adolescents / young adults and why this could be a strategy to help decrease HIV in this population (acceptance/feasibility/uptake studies in this age group?) (line 244)

**Response: Thank you for this suggestion. We have expanded the discussion on this topic, and we have added 2 references. Discussion, Page 15, Lines 266-268.**

4. Limitations: I would also mention that study does not account for potential ART resistance as a reason for lack of viral suppression

**Response: Thank you very much for your valuable comment, we have added this limitation to the section.**

***“Also, it is important to acknowledge that we did not specifically account for potential ART resistance as a reason for the lack of viral suppression in some individuals. ART resistance is a critical factor that can hinder the achievement of viral suppression among HIV-infected individuals”***

**Dsicussion, Page 16, line 288-290**

5. Please review for grammar.
- For example, acronym MoH for Ministries of Health in line 81 is later referred to as MOH in line 100 (make sure they are the same capitalization).

**Response: Thank you very much, we have reviewed carefully.**

- Sentence on line 106 is incomplete (ends with “included,” but doesn’t state what was included?).

**Response: Thank you for your valuable comment. We appreciate your feedback, and upon reviewing the sentence, we acknowledge its potential for confusion. To enhance clarity, we have removed the last part of the sentence.**

- Sentence on lines 234-235 is difficult to read, please try to reword: “However, African women through their reproductive live have repeated opportunities to be HIV tested which probably explains partially the increasing proportion of HIV diagnosis with age.” ◊ could consider changing to “However, African women have repeated opportunities to be HIV tested throughout their reproductive lives, which likely partially explains the increasing proportion of HIV diagnosis with age among women.”

**Response: Thank you very much, we have changed the sentence. *“However, African women have repeated opportunities to be HIV tested throughout their reproductive lives, which likely partially explains the increasing proportion of HIV diagnosis with age among women.”***

**Discussion: page 15, line 253-255**

- Make sure all acronyms are defined (PMTCT in line 239 should have a definition the first time it is used)

**Response: Thank you very much. We have included the definition.**
