# Supplementary material for: Adolescents and young adults are the most undiagnosed of HIV and virally unsuppressed in Eastern and Southern Africa: Pooled analyses from five population-based surveys
Source: PLOS Glob Public Health. 2023 Dec 22;3(12):e0002398. doi: 10.1371/journal.pgph.0002398 (PMC10745138; doi:10.1371/journal.pgph.0002398)
Supplement: Attachment — Submitted filename: Response to Reviewers.docx [file pgph.0002398.s007.docx]

**PGPH-D-23-00391R1
Adolescents and young adults are the most undiagnosed of HIV and virally unsuppressed in Eastern and Southern Africa: pooled analyses from five population-based surveys.
PLOS Global Public Health**


Thank you for addressing the reviewers' comments. This manuscript will be a valuable contribution to the literature.

**Response: Thank you very much for the review of our manuscript and your appreciation. We hope we have addressed the additional points raised in the revised version of the manuscript.**

Please note further revisions requested to bring the article into compliance with the journal's statistical policies and recommendations:

- please clarify the analysis is intended to be descriptive, and reword sentences such as 'To study the effect of age and gender in the HIV testing and treatment cascade' to more accurate language such as 'To describe the HIV testing and treatment cascade across age and gender groups'

**Response: Thank you for your comment. We have changed accordingly the text.**

**“To describe the HIV testing and treatment cascade across age and gender groups, we calculated the proportion of individuals diagnosed, treated and with viral load below 1,000 copies/mL according to their age (15-24, 25-34, 35-44, 45-59 years) and gender (women, men) separately”**

**Manuscript Page 7 Lines 126-127**

- please remove all mention of 'risk' in reference to odds ratios (or change the modeling approach to log binomial to calculate risk ratios instead of odds ratios)

**Response: Thank you for your valuable comment. We have reviewed and changed the sentences in the manuscript.**

**“Age and gender disparities within the HIV cascade of care are critical to focus interventions efficiently. We assessed gender-age groups at the highest probability of unfavorable outcomes in the HIV cascade in five HIV prevalent settings.”**

**Abstract Page 3 Line 37**

**“Compared to women 45-59 years, young people had a higher probability of being undiagnosed (men 15-24 years OR: 37.9, women 15-24 years OR: 12.2), untreated (men 15-24 years OR:2.2, women 15-24 years OR: 5.7) and virally unsuppressed (men 15-24 years OR: 1.6, women 15-24 years OR: 6.6).”**

**Abstract Page 3 Line 55**

**“We aimed to assess the age-gender groups with the lowest HIV diagnosis and treatment coverage and at the highest probability of unfavorable outcomes in the HIV cascade in five highly prevalent HIV settings.”**

**Manuscript Page 5 Line 87**

**“In addition, we estimated the odds of being undiagnosed, untreated and with viral load ≥1,000 copies/mL for several combined age-gender groups using the group of women 45-59 years as reference, in univariate logistic regression models”**

**Manuscript Page 7 Line 130**

**“Likelihood of being undiagnosed, untreated, and virally unsuppressed in combined gender-age groups.”**

**Manuscript Page 12 Line 205**

**“Men aged 15-24 years was the group with the highest likelihood (aOR=29) of being undiagnosed compared to women aged 45-59 years”**

**Manuscript Page 12 Line 207**

**“Women aged 25-34 years and men of older ages 35-59 years also had a higher likelihood of being undiagnosed compared to women 45-59 years though at a lower level (2-fold higher odds).”**

**Manuscript Page 12 Line 210**

**“Regarding the likelihood of being untreated among those diagnosed, women and men aged 15-34 years were the groups with the highest probability (2 to 3-fold higher odds) compared to women aged 45-59 years. These gender-age groups were also the ones with the highest probability of having viral load ≥1,000 copies/mL among those treated, except men 15-24 years for whom few individuals were included in this analysis.“**

**Manuscript Page 14 Lines 220-223**

**“In this large, pooled analysis of five community-based household surveys, adolescents and young adults had the lowest coverage in the HIV testing and treatment cascade, and the highest probability of being undiagnosed, untreated and virally unsuppressed after adjustment for sociodemographic factors and survey site”**

**Manuscript Page 14 Line 229**

**“Although few in our sample, men 15-24 years had a particularly high likelihood of being unaware of their HIV positive status compared to other age-sex groups”**

**Manuscript Page 14 Line 239**

**“Indeed, in our study, women 15-24 years were at a significantly increased probability to be unaware of their HIV status compared to older women.”**

**Manuscript Page 14 Line 249**

**“HIV-diagnosed young men and women had also an increased probability of being untreated and virally unsuppressed while on ART, implying that age differences persist along the HIV treatment cascade despite appropriate HIV diagnosis.”**

**Manuscript Page 15 Line 262**

- please strongly consider removing the adjusted multivariable modeling, which detracts rather than adds to this highly informative study, as the adjustment factors are much more likely to be mediating factors than confounders of the relationship between age/gender and these outcomes. The value of this study is in identifying the least served groups as laid out in the aims statement at the end of the introduction; the multivariable analysis is not in keeping with this aim. Please see the following paper for further details if helpful: Conroy S, Murray EJ. Let the question determine the methods: descriptive epidemiology done right. Br J Cancer. 2020;123(9):1351-1352. doi:10.1038/s41416-020-1019-z

**Response: Thank you for the suggestion and for the paper. We agree. We have removed the adjusted analyses and we have revised the text accordingly.**
